# Generation of High-Amylose Rice through CRISPR/Cas9-Mediated Targeted Mutagenesis of Starch Branching Enzymes

**DOI:** 10.3389/fpls.2017.00298

**Published:** 2017-03-07

**Authors:** Yongwei Sun, Guiai Jiao, Zupei Liu, Xin Zhang, Jingying Li, Xiuping Guo, Wenming Du, Jinlu Du, Frédéric Francis, Yunde Zhao, Lanqin Xia

**Affiliations:** ^1^Institute of Crop Sciences (ICS), Chinese Academy of Agricultural Sciences (CAAS)Beijing, China; ^2^China National Rice Research InstituteHangzhou, China; ^3^Functional and Evolutionary Entomology, Gembloux Agro-bio Tech, University of LiegeGembloux, Belgium; ^4^Section of Cell and Developmental Biology, University of California, San Diego, La JollaCA, USA

**Keywords:** amylose content (AC), CRISPR/Cas9, genome editing, resistant starch (RS), rice, starch, starch branching enzyme (SBE)

## Abstract

Cereals high in amylose content (AC) and resistant starch (RS) offer potential health benefits. Previous studies using chemical mutagenesis or RNA interference have demonstrated that starch branching enzyme (SBE) plays a major role in determining the fine structure and physical properties of starch. However, it remains a challenge to control starch branching in commercial lines. Here, we use CRISPR/Cas9 technology to generate targeted mutagenesis in *SBEI* and *SBEIIb* in rice. The frequencies of obtained homozygous or bi-allelic mutant lines with indels in *SBEI* and *SBEIIb* in T_0_ generation were from 26.7 to 40%. Mutations in the homozygous T_0_ lines stably transmitted to the T_1_ generation and those in the bi-allelic lines segregated in a Mendelian fashion. Transgene-free plants carrying only the frame-shifted mutagenesis were recovered in T_1_ generation following segregation. Whereas no obvious differences were observed between the *sbeI* mutants and wild type, *sbeII* mutants showed higher proportion of long chains presented in debranched amylopectin, significantly increased AC and RS content to as higher as 25.0 and 9.8%, respectively, and thus altered fine structure and nutritional properties of starch. Taken together, our results demonstrated for the first time the feasibility to create high-amylose rice through CRISPR/Cas9-mediated editing of *SBEIIb*.

## Introduction

Diet-related non-infectious chronic diseases, such as coronary heart disease, diabetes, and certain colon and rectum cancers, are major causes of morbidity and mortality in both developed and developing countries (Regina et al., 2006; Chen et al., 2012). Around 600 million people are estimated to live with diabetes in 2035 (Unwin et al., 2013). Resistant starch (RS) is a kind of starch or starch products that are not digestible and absorbed in the stomach or small intestine and passed on directly to the large intestine (Asp, 1992). A cereal grain higher in amylose content (AC) is always a good source of RS (Jiang et al., 2010). Cereals high in RS are benefit to improve human health and to reduce the risk of those serious non-infectious diseases (Regina et al., 2006). Consumption of RS could lead to a decreased glycemic index (GI), which represents the rise of blood sugar level following carbohydrate ingestion, and could also prevent the development of non-reversible insulin resistance in healthy individuals and diabetics, and thus decrease the incidence and mitigate the severity of type II diabetes (Giacco et al., 1998; Hoebler et al., 1999; Vonk et al., 2000). RS also produces short-chain fatty acids (SCFAs) which help to keep colon tissue healthier. The acetic, propionic, and butyric acids which composed the SCFA are beneficial to lower the intestinal pH, inhibit the growth of harmful bacteria, and encourage beneficial bacteria to grow in the bowel (Regmi et al., 2011). Although a high AC diet is highly desirable and healthier food for some consumers, cereal crops higher in AC are not widely available (Zhu et al., 2012). Thus, there is an increasing need to develop cereal crops high in RS to meet rapidly growing challenges in nutrition for public health at population level (Regina et al., 2006; Chen et al., 2012; Li and Gilbert, 2016).

Rice is one of the major staple food crops consumed by nearly half of the world population. Each year, about 4.5 million hectares of rice are grown worldwide. In Asia, rice and its processed products provide 60–70% daily calories for over 2 billion people (Food and Agriculture Organization [FAO], 2004). And about 90% of a milled rice kernel is starch. Starch consists of glucose polymers, amylose and amylopectin, which are polymerized through α-1, 4 and α-1, 6 linkages. Amylose has infrequent α-1, 6 linkages and a lower degree of polymerization (DP), whereas amylopectin is a large highly branched polysaccharide with higher DP. In cooked foods, amylose molecules re-associate rapidly to form a precipitate or gel upon cooling, forming complexes which resist digestion, whereas the re-associate rate of amylopectin molecules are slowly and thus the formed complexes are more readily digestible. Therefore, high-amylose products always have higher RS content (Regina et al., 2006). Amylose and amylopectin are synthesized via two different pathways. The synthesis of amylose requires an active granule-bound starch synthase (GBSS), whereas amylopectin is a product of a complex pathway which involves different isoforms of starch synthase (SS), starch branching enzymes (SBEs), and starch-debranching enzymes (SDBEs) (Ball and Morell, 2003). SBEs catalyze the cleavage of and transfer of α-1, 4-linked glucan chain and subsequent conjugate the part of that chain to the 6-hydroxyl groups along another chain, thus produce branches in amylopectin (Syahariza et al., 2013).

Analyses of the ACs of a set of germplasm collected by the International Rice Research Institute showed that AC in wild and cultivated rice ranges from 0 to 30% depending on the rice variety (Butardo et al., 2008). In rice endosperm, there exist at least three isoforms of SBEs, SBEI, SBEIIa, and SBEIIb. *SBEIIa* and *SBEIIb* share ∼80% sequence identity (Mizuno et al., 1992; Nakamura et al., 1992). *SBEIIa* is prodominantly expressed in leaves, while *SBEIIb* is mainly expressed in the rice grains (Yamanouchi and Nakamura, 1992; Ohdan et al., 2005; Yamakawa et al., 2007). SBEIIb is a major SBEII isoform in the rice endosperm (Yamanouchi and Nakamura, 1992). To further increase the AC, one can direct the starch synthesis toward amylose production through either overexpressing a suitable *GBSS* (*Waxy*) allele (Hanashiro et al., 1996; Itoh et al., 2003), or suppressing the expression of enzymes involved in amylopectin biosynthesis (Morell and Myers, 2005; Regina et al., 2006; Rahman et al., 2007). High amylose maize, wheat, and barley were achieved through down-regulation of *SBEII* (Boyer and Preiss, 1978; Satoh et al., 2003; Regina et al., 2006, 2010). In rice, high amylose *sbeIIb* mutants were generated by means of chemical treatment or radiation (Yano et al., 1985; Kim et al., 2005; Shu et al., 2006), or through hairpin RNA (hp-RNA) mediated RNA interference (RNAi) (Ossowski et al., 2008; Warthmann et al., 2008; Wei et al., 2010; Butardo et al., 2011). However, chemical or physical radiation induced mutations might be accompanied by undesired and uncharacterized mutations in the whole genome (Yano et al., 1985; Asaoka et al., 1986). Furthermore, RNAi-mediated interference of gene expression is often incomplete and the transgene expression varies in different lines. In addition, the transgenic lines are regarded as genetically modified and are subject to costly and time-consuming regulatory processes (Shan et al., 2015).

Genome editing technologies enable precise modifications of DNA sequences *in vivo* and offer a great promise for harnessing plant genes in crop improvement (Shan et al., 2013; Puchta and Fauser, 2014; Voytas and Gao, 2014; Li et al., 2015; Ma et al., 2015; Svitashev et al., 2015; Endo et al., 2016; Gao et al., 2016; Sun et al., 2016b). CRISPR/Cas9 (Clustered Regularly Interspaced Short Palindromic Repeats/CRISPR-associated Cas9 endonuclease) has revolutionized genome editing because of its specificity, simplicity, and versatility (Cong et al., 2013; Feng et al., 2014; Gao and Zhao, 2014; Zhou et al., 2014; Ma et al., 2015; Wang et al., 2015; Xie et al., 2015; Li J. et al., 2016; Paul and Qi, 2016; Sun et al., 2016a). The Cas9 protein functions as a nuclease and is directed to a target site by an engineered sequence-specific single guide RNA (gRNA). This site-specific targeting is determined by the first 20 nucleotides (target seed sequence) of the gRNA for the DNA recognition (Jinek et al., 2012). CRISPR/Cas9 induces site-specific double-strand breaks (DSBs), which can be then repaired by non-homologous end-joining (NHEJ) or homologous recombination (HR). NHEJ is error-prone and often introduces small insertions or deletions (indels) at cleavage sites, frequently generating frame-shifted knockout mutations (Jinek et al., 2012).

CRISPR/Cas9-mediated gene editing technology has the potential to greatly facilitate plant breeding. However, so far only a very few examples of improvement of agronomic important traits and creation of novel germplasm in crop plants have been reported (Ma et al., 2015; Svitashev et al., 2015; Li M. et al., 2016; Shi et al., 2016; Sun et al., 2016a,b). For example, through CRISPR/Cas9 mediated gene editing, substitution of P165 with Serine in the *ALS2* gene using either double-stranded DNA vectors or single-stranded oligonucleotides as repair templates yielded chlorsulfuron resistant maize plants (Svitashev et al., 2015). The replacement of a 5′ untranslated region of the *ARGOS8* gene by a native *GOS2* promoter resulted in increased grain yield by five bushels per acre under stress conditions in maize (Shi et al., 2016). In rice, targeted mutagenesis of rice *OsWaxy* (GBSS) gene by CRISPR/Cas9 led to the AC decreased from 14.6 to 2.6%, a phenotype similar to a natural glutinous rice variety (Ma et al., 2015). Mutants of *Gn1a, DEP1, GS3*, and *IPA1* genes, which function as regulators of yield parameters such as grain number, panicle architecture, grain size, and plant architecture, were generated through CRISPR/Cas9-mediated targeted mutagenesis, respectively (Li M. et al., 2016). In our previous study, multiple herbicide resistant rice plants were successfully achieved by CRISPR/Cas9-mediated HR for simultaneous *in planta* substitutions of two amino acid residues in acetolactate synthase (Sun et al., 2016b).

Here, we report the feasibility to create transgene-free high amylose rice plants through CRISPR/Cas9-mediated target gene editing of *SBEIIb* and *SBEI*. We defined the roles of *SBEI* and *SBEIIb* in determining the AC, fine structure of amylopectin, and physiochemical properties of starch. This work enables the improvement of nutritional properties of starch in rice grain, thus potentially providing health benefits to many people.

## Materials and Methods

### Construction of the CRISPR/Cas9-Related Vectors

The vectors used in this study were based on the vector pCXUN-Cas9 in which the codon-optimized *Cas9* was driven by the ubiquitin gene promoter of maize (*Zea mays* L.). The backbone of pCXUN-Cas9 contains a hygromycin resistant gene (*hptII*) for callus selection. The *Pme*I in pCXUN-Cas9 were used for introducing the gRNAs expression cassettes.

The gRNA expression cassettes were synthesized by overlapping PCR. Target-specific sequence of gRNA1 (target *SBEI*) or gRNA2 (target *SBEIIb*) were put at the 5′-end of primers SBEI-F1/SBEI-R1 and SBEIIb-F1/SBEIIb-R1, respectively. Because OsU3 promoter sequences were used in this experiment, we also placed an A at the first nucleotide of the target sequence. Two PCR reactions were performed by using the plasmid pOsU3-gRNA as a template. The first PCR was performed using primer set hrpme-u3F/SBEI-R1, and the second one used primer set SBEIIb-F1/hrpme-u3R (Supplementary Table S2). Products of PCR 1 and 2 were used as templates for the third PCR reaction with the primer set hrpme-u3F/hrpme-u3R to generate the full length gRNA1 fragment (Supplementary Table S2). The same procedure was used to obtain the gRNA2 fragment by using the primer sets SBEIIb-F1, hrpme-u3R, and hrpme-u3F and SBEIIb-R1 (Supplementary Table S2). At the 5′-end of the primer pairs of hrpme-u3F/ hrpme-u3R, the sequences are homologous to the sequences outsides of *Pme* I sites in pCXUN-Cas9, respectively. Then, gRNA1 and gRNA2 fragments were cloned to the linearized pCXUN-Cas9 with *Pme* I, by using pEASY-Uni Seamless Cloning and Assembly Kit (TransGen Biotech, Beijing, China).

### *Agrobacterium-*Mediated Rice Transformation

The vectors described above were transferred into *Agrobacterium tumefaciens* strain EHA105 by electroporation. Transformation of rice calli was performed as described by Hiei et al. (1994). The calli derived from *japonica* cv. Kitaake were selected on media containing 50 mg/L hygromycin for 4 weeks. Then the vigorously grown calli were transferred to regeneration media to generate green plants.

### Molecular Characterization of the Mutant Plants

Rice genomic DNA was extracted by using a DNA Quick Plant System (TransGen Biotech, Beijing, China). 50 ng of genomic DNA were used as a template to perform PCR amplification using Taq polymerase (Tiangen, Beijing, China). The primer sets RC11-F/RC11-R and RC33-F/RC33-R were designed to flank the designated target sites. The PCR products amplified by the primer sets RC11-F/RC11-R and RC33-F/RC33-R were digested with *Cac*8 I and *Dde* I, respectively. Then the undigested band was recovered and directly sequenced to screen for the plants with mutations in *SBEI* or *SBEIIb*. The sequence chromatograms were analyzed by a web-based tool^1^ to check the genotype and zygosity of the tested plants. PCR products were also cloned into the TA cloning vector P-easy (TransGen Biotech, Beijing, China), and 10 positive colonies were sequenced for each sample. The PCR primer sets used for the PCR/RE assay and detection of the presence of Cas9 and gRNA were as listed in Supplementary Table S2. T_1_ homozygous *sbeI* and *sbeIIb* mutant lines derived from either T_0_ homozygotes or bi-allelic lines were subjected to further analyses.

### Measurement of Grain Composition and Molecular Structure of Starch

The harvested mature panicles were dried at 37°C for at least 3 days. Thousand-grain weights (g) of selected lines were weighed in triplicate. The SeedCount (SeedCount, Australasia, Pty, Ltd) was used to determine grain appearance and dimensions. The opacity of seeds was investigated using the chalkiness index following the standard of Australian rice industry. Leitz M8 stereomicroscope was used to take photomicrographs of whole brown grain samples.

The granular morphology of starch was investigated by scanning electron microscopy (SEM). Grain samples were transversely cut and mounted on circular aluminum stubs with double-sided sticky tape. Samples were examined and photographed by FEI Quanta 450 (FEI Company, Hillsboro, OR, USA).

The total starch, AC and RS in the brown rice flour were measured with the starch assay kits Megazyme K-STAR, K-AMYL and K-RSTAR (Megazyme, Wicklow, Ireland)^2^.

To analyze the chain length distributions (CLDs) of amylopectin, 25 mg of rice powder was digested with isoamylase (Megazyme, Wicklow, Ireland^2^) at 37°C for 24 h for release of the N-linked oligosaccharides. After release, the oligosaccharides were labeled with a fluorophore called 8-aminopyrene-1, 3, 6, 6-trisulfonate. For measurement of the stoichiometry, each labeling reaction is composed of one APTS molecule per molecule of oligosaccharide. Added maltose quantitative control/mobility marker and then analyzed by capillary electrophoresis (PA800 plus pharmaceutical analysis system, Carbohydrate labeling and analysis, Beckman Coulter, America)^3^.

### Measurement of Pasting and Gelatinization Properties

Three grams of brown rice flour (0.5 mm or less, 14% moisture basis) was put in 25 ml of distilled water and mixed. The sample was measured by Rapid Visco Analyzer (RVA Techmaster, Newport Scientific, Narrabeen, NSW, Australia), according to the America Association of Cereal Chemists (AACC) Approved Method 61-02.01 (AACC, 1995). The gelatinization of rice flour in urea solutions was determined as described by Nishi et al. (2001).

The sample which finished RVA analysis was cool down to room temperature, then stored at 4°C and relative humidity 75% for 24 h for retrogradation. The retrograded cooked rice flour was performed texture profile analysis (TPA) analysis. The hardness, gumminess and chewiness were measured using a texture analyzer with 25N load cell with a two-cycle compression (TMS-Pro, Food technology corporation, American). The deformation level was 70% of original sample height and the partly broken rice was compressed again.

For the gelatinization temperature and enthalpy analysis, 5 mg of brown rice flour was mixed with 10 μL of distilled water and placed inside an aluminum sample cup, and sealed, and then the samples were analyzed by a differential scanning calorimeter (DSC1 STARe system, METTLER TOLEDO, Switzerland).

### Measurement of Reducing Sugar and Pentosan

The *in vitro* digestibility of rice flour was determined as described by Englyst et al. (1992) with minor modifications. The rice flour 50 mg weighed in screw-capped tubes, 4 ml of sodium acetate buffer (0.5 M, pH = 5.2) and 1 ml (3 U/ml) pancreatic α-amylase (Megazyme, Wicklow, Ireland) were added to each tube. The sample was incubated in a shaking water bath (200 rpm) at 37°C. Aliquots (0.5 ml) were taken at intervals and mixed with 0.5 ml (1.2 M) of acetic acid to terminate the reaction. After centrifugation (2000 rpm, 10 min), the reducing sugar content released in the supernatant from each sample was measured using 3, 5-dinitrosalicylic acid. Total pentosan content was examined as described by Butardo et al. (2011).

### Statistical Analysis

Grain morphology and starch physiochemical properties of different mutant lines were analyzed by using a one-way variance analysis (ANOVA). The differences were examined using a Student’s *t*-test. Significance (*P*-value) was evaluated at the 1 or 5% level for all comparisons. For each treatment, the standard error of the mean (SEM) was calculated based on at least three biological replicates. For the χ^2^ test, *P* > 0.5 were considered to be very good agreement with expected segregation ratio.

## Results

### CRISPR/Cas9-Mediated Targeted Mutagenesis of *SBEI* and *SBEIIb* in Rice

**FIGURE 1:**
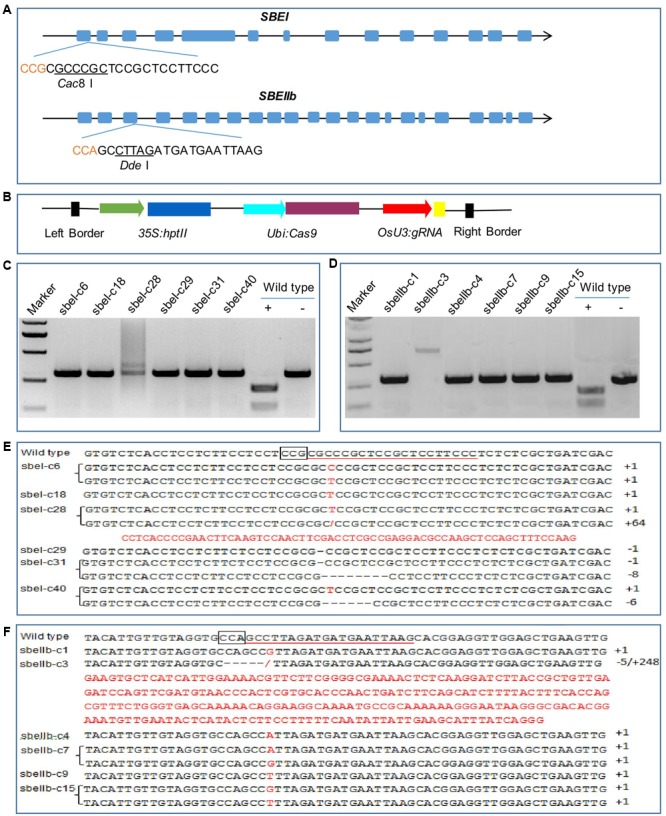
**CRISPR/Cas9 induced mutations in the *SBEI* and *SBEIIb* genes. (A)** A schematic map of the gRNA target sites on the genomic regions of *SBEI* and *SBEIIb.* Introns are shown as lines; exons shown as boxes; The PAM motif (CCN) is shown in red; the recognition sites of the enzymes *Cac*8 I and *Dde* I are underlined. **(B)** Schematic presentation of the T-DNA structure in CRISPR/Cas9-mediated genome editing construct. **(C,D)** Detection of mutations in *SBEI* and *SBEIIb* via PCR/RE assay in T_0_ generation. The PCR products of *sbeI* and *sbeIIb* mutant lines are resistant to *Cac*8 I and *Dde* I digestion, respectively. As for wild type, ‘+’ means the PCR products of wild type DNA digested by *Cac*8 I or *Dde* I; ‘-’ means without digestion. **(E,F)** Sequencing results of the *sbeI* and *sbeIIb* mutant lines, the PAM motifs are boxed, target sequences are underlined, insertions are highlighted in red, dashes indicate deletions. The *sbeI-c18, sbeI-c29, sbeIIb-c1, sbeIIb-c3, sbeIIb-c4*, and *sbeIIb-c9* are homozygous lines; the *sbeI-c6, sbeI-c28, sbeI-c31, sbeI-c40, sbeIIb-c7*, and *sbeIIb-c15* are bi-allelic lines.

We targeted the first exon of *SBEI* (GenBank Accession no GQ150904.1) and the third exon of *SBEIIb* (GenBank Accession no GQ150916.1) (**Figure 1A**). Both targets contained restriction sites that were used for screening mutations using PCR-based restriction enzyme (PCR/RE) digestion assay (**Figure 1A**). We placed the gRNA cassette into the binary vector pCXUN (**Figure 1B**). We transformed the CRISPR/Cas9 vectors into rice embryogenic calli through *Agrobacterium*-mediated transformation, and generated transgenic lines after two rounds of hygromycin selection. In total, we obtained 32 *sbeI* mutant rice plants from 40 hygromycin-resistant transgenic plants. Our PCR/RE assay and sequence analyses indicated that, of these 40 transgenic plants, 12.5, 27.5, and 40.0% were heterozygous, bi-allelic, and homozygous *sbeI* mutant lines, respectively (**Figures 1C,E** and **Table 1**). For *SBEIIb*, 21 *sbeIIb* mutant plants were recovered from 30 independent transgenic plants regenerated, with a frequency of 6.7, 36.6, and 26.7% for heterozygous, bi-allelic, and homozygous lines, respectively (**Figures 1D,F** and **Table 1**). Consistent with previous CRISPR/Cas9 studies (Feng et al., 2014), we found that the majorities of the mutations identified in the mutant lines were small deletions or insertions near the target sites (**Figures 1E,F** and **Table 1**). However, some mutants harbored large insertions such as the *sbeI-c28* and *sbeIIb-c3* mutant lines, which had a 64 and 248 bp insertion at the target sites, respectively (**Figures 1E,F**). Interestingly, the inserted sequences were derived from the vector used in this study in these both cases.

**Table 1:** CRISPR/Cas9-mediated target mutations in *SBEI* and *SBEIIb* in rice.

Target gene	No. of T_0_ plant tested	Non-edited plants (%)	Zygosity		
			Heterozygote (%)	Bi-allelic (%)	Homozygote (%)
*SBEI*	40	8 (20.0)	5 (12.5)	11 (27.5)	16 (40)
*SBEIIb*	30	9 (30.0)	2 (6.7)	11 (36.7)	9 (26.7)

We further investigated if off-target effects occurred in our experiments. Based on the predictions of the CRISPR-P tool^4^, we identified the potential off-target sites for *SBEI* and *SBEIIb* genes. We used site-specific genomic PCR and Sanger sequencing to determine whether the predicted off-target sites were also edited. As shown in Supplementary Table S1, no mutations were detected at the putative off-target loci in rice genome in T_0_ and T_1_ offspring of these obtained mutant lines.

### Inheritance and Stability of the Identified Mutations and Generation of Transgene-Free Mutant Rice Lines

To investigate whether the mutations generated through CRISPR/Cas9-mediated editing were heritable, we analyzed the segregation patterns at T_1_ generation. T_0_ mutant plants were self-pollinated and the resulting T_1_ plants were used for segregation analysis (**Table 2**). We randomly selected 15 to 37 T_1_ progenies derived from each T_0_ plant for genotyping analysis (**Table 2**). We found that all of the mutations detected in T_0_ plants were transmitted to the T_1_ generation without the occurrence of new mutations (data not shown). All T_1_ plants from T_0_ homozygotes were homozygous for the same mutations. Bi-allelic mutations in T_0_ plants were apparently transmitted to T_1_ generation in a Mendelian fashion. For example, *sbeI-c31* was a bi-allelic line with a 1 bp deletion (d1) and an 8 bp deletion (d8). Among the 37 T_1_ plants analyzed, 9 contained d1, 17 were bi-allelic (d1/d8), and 11 were d8, consistent with the predicted Mendelian segregation (χ^2^ test, *P* > 0.05). We also noticed some rare segregation patterns. For example, mutations in *sbeIIb-c3* showed a non-Mendelian segregation ratio of 1:1, which may be caused by aberrant gamete or seed formation.

**Table 2:** Transmission and segregation of CRISPR/Cas9-mediated target mutagenesis and transgenes from T_0_ to T_1_ generation.

T_0_ line	T_0_ plants				Segregation of mutations and T-DNA in T_1_ progenies			
	Genotype	Zygosity	Cas9/gRNA/ hptII	Number of T_1_ plants tested	Targeted mutations	Expected segregation ratio	*P*-value	Cas9/gRNA/hptII
*sbeI-c6*	i1a/i1b	Bi-allelic	+	26	6 i1a, 12 i1a/i1b, 8 i1b	1:2:1	0.97	24+ : 2-
*sbeI-c18*	i1	Ho	+	15	15 i1	N.A	N.A	15+ : 0-
*sbeI-c28*	i1/i64	Bi-allelic	+	34	17 i1, 11 i1/i64, 6 i64	1:2:1	0.64	26+ : 8-
*sbeI-c29*	d1	Ho	+	15	15 d1	N.A	N.A	15+ : 0-
*sbeI-c31*	d1/d8	Bi-allelic	+	37	9 d1, 17 d1/d8, 11 d8	1:2:1	0.98	28+ : 9-
*sbeI-c40*	i1/d6	Bi-allelic	-	35	10 i1, 17 i1/d6, 8 d6	1:2:1	0.99	0+ : 35-
*sbeIIb-c1*	i1	Ho	+	22	22 i1	N.A	N.A	17+ : 5-
*sbeIIb-c3*	d5/i248	Ho	+	38	38 (d5/i248)	N.A	N.A	17+ : 21-
*sbeIIb-c4*	i1	Ho	+	17	17 i1	N.A	N.A	17+ : 0-
*sbeIIb-c7*	i1a/i1b	Bi-allelic	+	36	9 i1a, 20 i1a/i1b, 7 i1b	1:2:1	0.96	36+ : 0-
*sbeIIb-c9*	i1	Ho	+	15	15 i1	N.A	N.A	15+ : 0-
*sbeIIb-c15*	i1a/i1b	Bi-allelic	+	19	5 i1a, 8i1a /i1b, 6 i1b	1:2:1	0.95	18+ : 1-

*T_0_ plants can be bi-allelic, homozygote (Ho) and heterozygote (He). In d#, the # refers to the number of bp deleted from the target site; i#a or i#b, # refers to the number of bp inserted at target sites with different nucleotide or different positions (a or b). According to the χ^2^ test, *P* > 0.5, means in very good agreement with expected Mendelian segregation ratio; N.A, not applicable. For transgene analysis, ‘+’ represents that *Cas9/gRNA/hptII* was detected and ‘-’ shows *Cas9/gRNA/hptII* not detected. Some of the T_0_ lines such as *sbe-c1* had only one T-DNA insertion as demonstrated by around 1/3 of *Cas9/gRNA/hptII*-free plants in T_1_ progenies, whereas *sbeI-c6* T_0_ plant probably had two T-DNA insertions because only about 1/12 of T_1_ plants were *Cas9/gRNA/hptII* free. The failures to identify *Cas9/gRNA/hptII*-free plants at T_1_ generation for some lines were probably due to either multiple insertions or tight linkage to the mutations.*

**FIGURE 2:**
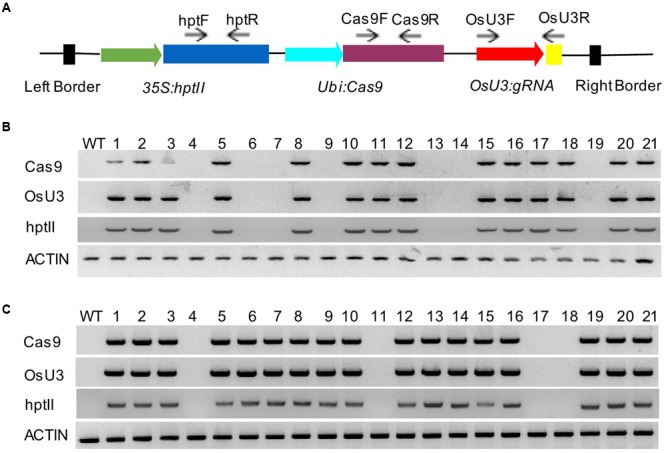
**Isolation of transgene-free T_1_ plants from the *sbeI-c31* and *sbeIIb-c1* mutant lines. (A)** A schematic description of the location/direction of the primer sets used for the analysis for the presence of T-DNA sequences in T_1_ plants of *sbeI-c31* **(B)** and *sbeIIb-c1* lines **(C)** T_0_ lines may contain CRISPR/Cas9 complex in their genome. However, self-pollination of T_0_ lines allows the removal of the T-DNA following segregation in the T_1_ generation plants. DNA fragments of *OsU3:gRNA, Cas9* and *hptII* were not detected in some T_1_ plants of *sbeI-c31* lines (Number 4, 6, 7, 13, 14, and 19) **(B)** and T_1_ plants of *sbeIIb-c1* (Number 4, 11, 17 and 18) **(C)**, respectively. The control PCR product was amplified from the endogenous *ACTIN* gene, indicating that the genomic DNAs used have sufficient quality for PCR. WT, wild type DNA control.

To determine whether plasmid DNA sequences, especially *Cas9, gRNA*s, and *hptII* sequences, were present in these mutant lines, we performed PCR amplification using the primer sets designed to specifically amplify *Cas9, gRNA*s, and *hptII* sequences, respectively (**Figure 2A** and Supplementary Table S2). Among the 12 T_0_ mutant lines analyzed, 11 contained *gRNA*s and *Cas9* sequences. We were able to recover transgene-free mutants at T_1_ generation. Interestingly, *sbeI-c40* mutant line did not contain *Cas9, gRNA*, and *hptII* sequences at both T_0_ and T_1_ generations, suggesting that this line escaped from hygromycin selection and the mutations in this line were generated through transient expression of *Cas9/gRNA*. We also noticed that the indels in *sbeIIb-c3* mutant line was homozygous and was caused by an insertion of part of the pCXUN-Cas9 backbone sequences; therefore, these plants are not suitable for future applications. *SbeI-c28* was a bi-allelic line with a 1 bp deletion (d1) and a 64 bp insertion (i64) which was derived from pCXUN-Cas9 backbone sequences. However, we were able to recover homozygous plants with only 1 bp of deletion and transgene-free T_1_ plants (**Table 2**). Among the rest nine T_0_ lines tested, *Cas9/gRNA* and *hptII* transgene-free plants were successfully recovered from four T_0_ mutant lines such as two lines for *SBEI* (*sbeI-c6* and *sbeI-c31*) and another two lines for *SBEIIb* (*sbeIIb-c1* and *sbeIIb-c15*), in the T_1_ generation following segregation (**Figures 2B,C** and **Table 2**), indicating that transgene-free mutants could be generated with targeted mutagenesis in *SBEI* and *SBEIIb* in rice even in a single generation. More transgene-free plants will be expected in the following generations given that the transgene will segregate following a Mendelian model.

### Effects of *sbe* Mutations on Morphology of Grain and Starch Granule

**FIGURE 3:**
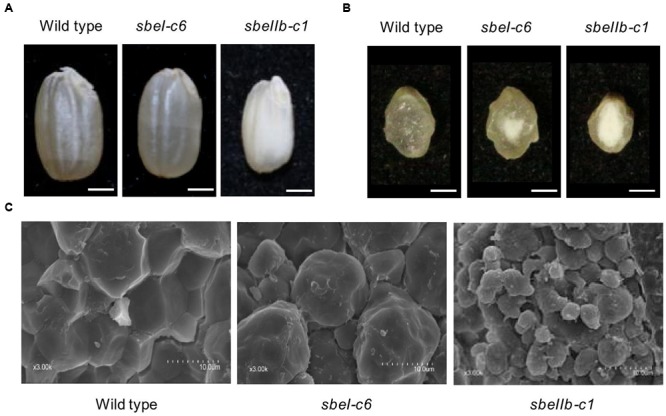
**Grain and starch granule morphology profiles of the *sbeI* and *sbeIIb* mutant lines. (A)** Morphologies of the brown grains. Bars = 1 mm. **(B)** Transverse sections of grains from wild type, *sbeI-c6* and *sbeIIb-c1* mutant lines. Bars = 1 mm. **(C)** Scanning electron microscopy images of the starch in wild type, *sbeI-c6* and *sbeIIb-c1* mutant grains. Bars = 10 μm.

Brown grains of 12 T_1_ homozygous *sbeI* and *sbeIIb* mutant lines derived from either T_0_ homozygotes or bi-allelic lines were used to analyze the grain and starch granule morphology. Brown grains of *sbeIIb* mutant lines were opaque throughout, while the *sbeI* mutant lines were chalky. In contrast, grains of wild type appeared uniformly translucent (**Figures 3A,B**). The weight, width, and thickness of grains from *sbeIIb* mutant lines were significantly lower than those of wild type, whereas no significant differences were observed between *sbeI* mutant lines and wild type control (**Table 3**). It is worth to mention that we did not observe obvious starch granule morphological changes in the grains of T_1_ homozygous plants of *sbeI-c40* with 6 bp deletion (d6), which were progenies from T_0_ *sbeI-c40* bi-allelic line with i1/d6 mutations, indicating that the in-frame deletion of six nucleotides is likely to still produce functional SBEI protein. However, obvious alternations in both the appearance of brown grains and morphology of the starch granules in comparison with the wild type were observed in a T_1_ homozygous line *sbeIIb-c3* with d5/i248 mutations (**Figure 1F**), probably because the large in-frame indels (deletions or insertions) disrupted the function of target gene.

**Table 3:** Grain morphology and starch physiochemical properties of different mutant lines.

Properties	Wild type	*sbeI-c6* (i1a/i1a)	*sbeI-c31* (d8/d8)	*sbeIIb-c1* (i1a/i1a)	*sbeIIb-c15* (i1b/i1b)
Thousand-grain weight (g)	24 0.42 a	24 0.45 a	24 0.13 a	17 0.46 b	17 0.36 b
Length (mm)	5.5 0.10 a	5.5 0.10 a	5.4 0.10 a	4.9 0.10 b	4.8 0 b
Width (mm)	2.5 0.10 a	2.5 0.10 a	2.4 0.10 a	2.0 0 b	2.1 0 b
Thickness (mm)	2.0 0.01 a	2.0 0.10 a	2.0 0.10 a	1.8 0 b	1.7 0 b
Chalkiness (% per grain)	0-10	0-10	0-10	75-100	75-100
Hardness	1.26 0.01 a	1.04 0.02 b	1.10 0.01 b	0.30 0.01 c	0.31 0.01 c
Gumminess	1.10 0.05 a	0.73 0.06 b	0.76 0.09 b	0.27 0.03 c	0.29 0.02 c
Chewiness	8.25 0.01 a	6.52 0.04 b	6.69 0.06 b	0.15 0.04 c	0.22 0.02 c
Gel consistency	31 1	28 1	28.9 1	33.2 0.6	33.1 0.2
Onset gelatinization temperature (°C)	60 0.40 a	55 0.61 a	56.5 1.10 a	73.6 0.92 b	74.5 1.20 b
Peak gelatinization temperature (°C)	66.9 0.80 a	62.4 1.20 a	63.6 0.70 a	80.4 1.40 b	81.2 2.20 b
End gelatinization temperature (°C)	73.9 1.30 a	70.1 1.11 a	71.2 0.80 a	86.7 1.50 b	88.1 1.80 b
Gelatinization enthalpy (J/g)	4.5 0.12 a	2.68 0.06 b	2.58 0.07 b	6.6 0.10 c	6.7 0.12 c
Pentosans (%)	1.72 0.10 a	1.78 0.02 a	1.77 0.015 a	2.14 0.08 b	2.22 0.04 b

*Values reported are mean ± SEM. Mean values with different letters are significantly different. Number and characters indicated in the brackets after the name of each mutant line, i1 and d8, represent insertion of 1 bp or deletion of 8 bp at the target sites, respectively, and i1a or i1b indicates the 1 bp insertion of different nucleoid base.*

We also analyzed the morphology of starch granules using SEM. Starch granules from *sbeIIb* mutant lines were heterogeneous in size and shape, with large spaces in between, and without compound granular organization. The starch granules from *sbeI* lines were big, rounded, and irregularly arranged. In contrast, the starch granules of wild type were compact, compound, and angular (**Figure 3C**). Changes in starch granule morphology were more pronounced in *sbeIIb* mutant lines than that in *sbeI* lines. The surfaces of both wild type and the *sbeI* mutant granules were smooth with no or few structural debris (**Figure 3C**).

### Impact of *sbe* Mutations on Starch Structures

**FIGURE 4:**
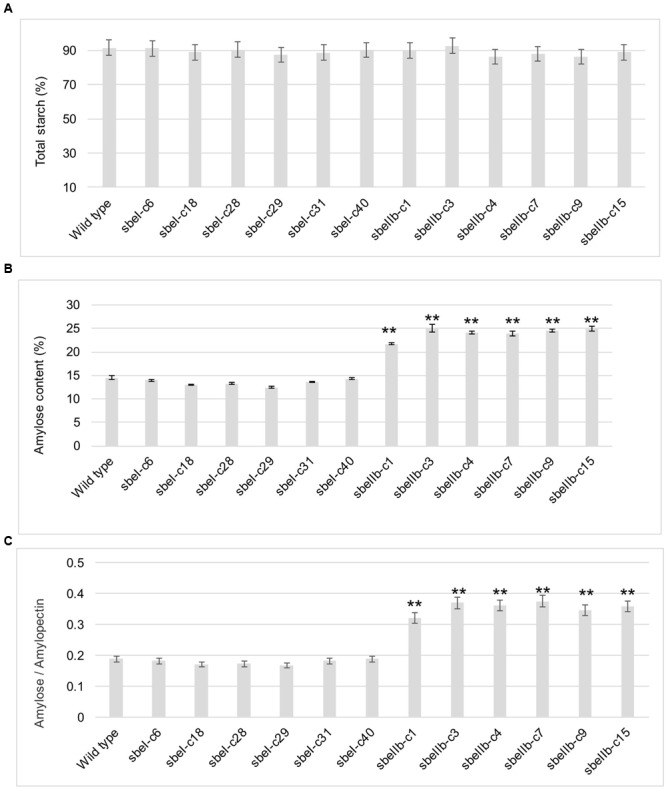
**The starch contents of polished grains from different *sbeI* and *sbeIIb* mutant lines. (A)** Total starch contents of polished grains of different *sbeI* and *sbeIIb* mutant lines. **(B)** Amylose contents (ACs) of polished grains from different mutant lines. **(C)** Ratios of amylose/amylopectin of the starch in different mutant lines (^∗∗^Student’s *t*-test, *n* = 3, *P* < 0.01).

**FIGURE 5:**
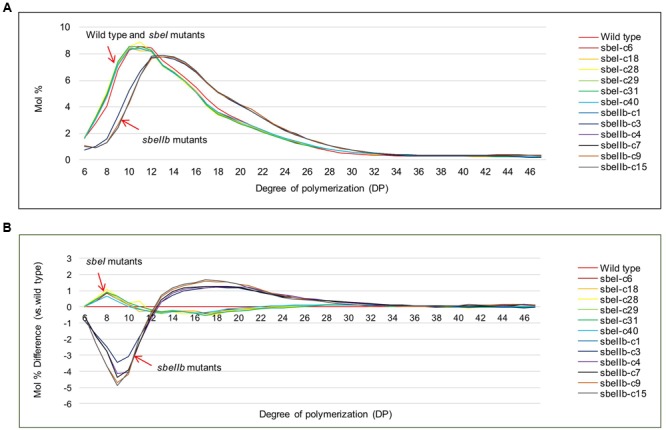
**Chain-length distribution profiles of isoamylase debranched starches from different mutant lines. (A)** Chain length distribution (CLD) profiles of debranched starch from *sbeI* and *sbeIIb* mutant lines compared with wild type. **(B)** Differences were calculated by subtracting chain-length distributions of isoamylase-debranched wild type from *sbeI* and *sbeIIb* mutant lines, respectively.

The total starch contents in the polished grains of both *sbeI* and *sbeIIb* mutant lines were comparable to that of the wild type (**Figure 4A**). However, *sbeIIb* mutant lines produced grains with much more amylose compared to *sbeI* mutant lines and wild type (25 vs. 15%) (Student’s *t*-test, ^∗∗^*P* < 0.01) (**Figure 4B**). The ratio of amylose/amylopectin was also increased significantly in *sbeIIb* mutant lines (Student’s *t*-test, ^∗∗^*P* < 0.01) (**Figure 4C**). More importantly, all *sbeIIb* mutant lines displayed altered CLDs of debranched starches. The grains of *sbeIIb* mutant lines had a significant increase in the ratio of amylopectin longer chains with DP higher than 14 (DP > 14) and a concomitant decrease in the short chains (DP 6–12). For the *sbeI* mutant lines, we observed a slight increase in the ratio of amylopectin short chains (DP 6–12) followed by a decrease in the longer chains (DP > 14), when compared with the wild type control (**Figure 5**). The decrease of short chains was pronounced in *sbeIIb* mutant lines (>3% for DP 9), whereas the increase reached up to 1% for DP 18 (**Figure 5B**).

### Physicochemical and Nutritional Properties of Starch

**FIGURE 6:**
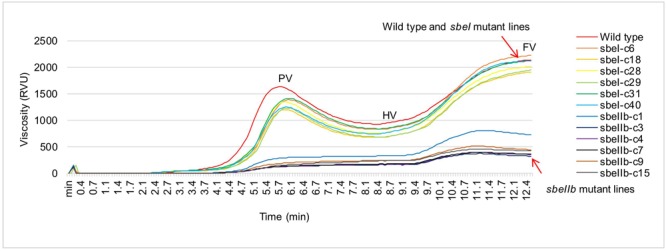
**Rapid viscosity profiles of rice flours from different *sbe* mutant lines.** Pasting curves of the flours from *sbeI* and *sbeIIb* mutant lines were measured by a rapid visco analyser (RVA). PV, peak viscosity; HV, hot viscosity; FV, final viscosity.

The changes of starch granule morphology and fine structure of amylopectin resulted in the alternations of physicochemical and nutritional properties of starch. In comparison with the wild type control, the polished grains of *sbeI* and *sbeIIb* mutant lines showed a significantly decrease in hardness, gumminess, and chewiness with more pronounced effects observed in the *sbeIIb* mutant lines (**Table 3**). Furthermore, the flours from *sbeIIb* mutant lines failed to paste at the temperature profile set for RVA analysis, and demonstrated significant decreased values of several viscosity parameters such as the peak viscosity (PV), hot viscosity (HV), breakdown value (BDV, PV-HV), final viscosity (FV) and setback value (SBV, FV-HV). Whereas those values of the *sbeI* mutants were similar to those of wild type with only a slight decrease of PV (**Figure 6** and **Table 3**). We further analyzed the thermal properties of starch from both *sbeI* and *sbeIIb* mutant lines by differential scanning calorimetry (DSC). The peak temperature indicates the transition induced by melting amylopectin crystallities, whereas the end temperature represents the transition of the dissociation of the amylose-lipid complex (Regina et al., 2010). As indicated in **Table 3**, the onset, peak and end gelatinization temperatures, and gelatinization enthalpy of thermo-gelatinization for the starch of *sbeIIb* mutant rice lines were significantly increased compared to those of the wild type (Student’s *t*-test, ^∗∗^*P* < 0.01). In contrast, the *sbeI* mutant starches had similar gelatinization temperatures and decreased gelatinization enthalpy of thermo-gelatinization compared to those of the wild type (Student’s *t*-test, ^∗∗^*P* < 0.01) (**Table 3**). These results indicated that the flours of the *sbeIIb* mutant rice lines possessed a stronger resistance to alkaline gelatinization and swelling of its granules.

**FIGURE 7:**
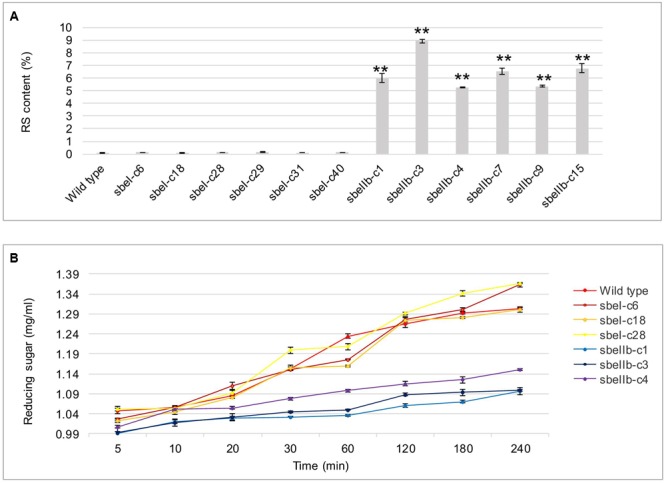
**Contents of resistant starch and reducing sugar in the grains from different *sbe* mutant lines. (A)** RS content of grains of mutant lines and wild type. **(B)** Content of reducing sugar after a-amylase treatment (^∗∗^Student’s *t*-test, *n* = 3, *P* < 0.01).

The *in vitro* RS content of freshly cooked *sbeIIb* mutant grains reached as high as 9.8% when measured following the protocol described by the AOAC Method 2002.02 (McCleary et al., 2002), which is significantly higher than that of wild type and *sbeI* mutant lines in which little or no RS was detected in the grains (**Figure 7A**). Furthermore, the content of reducing sugar, which is positively and linearly correlated to GI, was much lower in the grains of *sbeIIb* mutant lines, while no obvious difference was detected between the *sbeI* mutant lines and wild type (**Figure 7B**). At the same time, the content of pentosans, which are perceived as one of the major components of healthy dietary fiber, was also significantly higher in the grains of *sbeIIb* mutant lines compared to wild type, whereas no obvious difference was observed between *sbeI* mutant lines and wild type (**Table 3**).

## Discussion

In this study, we successfully generated high amylose rice by CRISPR/Cas9-mediated targeted mutagenesis in *SBEIIb*. We designed gRNAs specifically targeting *SBEI* and *SBEIIb* in rice and obtained transgene-free homozygous *sbeIIb* mutants with a significantly increased AC and RS contents (**Figure 7**). Compared to conventional breeding, chemical or physical radiation induced mutagenesis and transgene-based strategies, CRISPR/Cas9-mediated genome editing technology can modify a target gene more precisely in a very convenient way (Jones, 2015). No need for laborious crossing and backcrossing and ‘clean’ plants without transgene could be obtained in one or two generations. Thus, by using a *japonica* rice variety *Kitaake* as an example, we here further demonstrated the advantages of CRISPR/Cas9-mediated genome editing in crop improvement and presented a better alternative strategy to breed for high amylose rice to meet increasing demand of people who suffer from diet-related non-infectious chronic diseases.

Reduction of SBEs’ activities led to decreased ratio of branch points in the amylopectin fraction and increased AC (Morell et al., 2004). Another effect is on the frequency or distribution of branches (Thompson, 2000; Yao et al., 2004). Whereas *SBEIIa* and *SBEIIb* usually generate short chains, *SBEI* has an apparent function in the formation of long chains of amylopectin (Nishi et al., 2001; Nakamura, 2002; Satoh et al., 2003). Different SBEs play different roles in starch biosynthesis in different plant species. No apparent functionof SBEI was foundin starch metabolism in *Arabidopsis* (Dumez et al., 2006) and abolishing SBEI activity resulted in no measurable impact on starch structure or functionality in potato, maize, and wheat (Safford et al., 1998; Blauth et al., 2002; Regina et al., 2004). In contrast, a significant increase of AC content was achieved through down-regulation or elimination of *SBEII* expression in different crop species. For example, down-regulation of *SBEII* in potato leads to an increase in amylose to approximately 35% (Jobling et al., 1999), whereas potato starch with an AC of 70% or higher was yielded by co-suppression of *SBEI* and *SBEII* (Schwall et al., 2000). In barley, suppression of both *SBEIIa* and *SBEIIb* generated starch with AC higher than 70% (Regina et al., 2010). In barley, starch granules containing almost entirely amylose were obtained by suppression of all the SBE genes (Carciofi et al., 2012). In maize endosperm, the amylose extender (ae) phenotype with ACs ranged from 50 to 80% were obtained by lesions in the *SBEIIb* gene (Garwood et al., 1976), whereas lesions in *SBEIIa* had no effect on endosperm starch (Blauth et al., 2001). In wheat, suppression of the expression of *SBEIIa* yielded a >70% AC (Regina et al., 2006). In rice, down-regulation of *SBEIIb* resulted in increased AC up to 25–30% in a *japonica* background (Mizuno et al., 1993; Nishi et al., 2001). Artificial microRNA and hairpin RNA-mediated RNAi silencing of *SBEIIb* expression in rice grains resulted in apparently elevated levels of amylose and RS and thus lower GI values (Butardo et al., 2011). Inhibition of *SBEI* led to unaltered AC. In contrast, the *SBEIIb* RNAi transgenic rice grains showed a substantial increase in AC. However, the increase of AC was much more significant in both *SBEI* and *SBEIIb* antisense RNAs transgenic grains, indicating that simultaneous manipulation of *SBEI* and *SBEIIb* genes has the potential to dramatically increase the AC in rice grains (Zhu et al., 2012). Our results are consistent with previous studies. Knock-out of *SBEIIb* leads to the increase of AC and longer amylopectin chains (**Figures 4**, **5**). In contrast, *sbe1* mutant lines and wild type were very similar in terms of AC content (**Figure 4**) (Mizuno et al., 1993; Nishi et al., 2001; Butardo et al., 2011; Zhu et al., 2012). The *sbeIIb* mutant lines contained more long chains than wild type, with increased proportion of the long chains (DP > 14) and a concomitant decrease in the ratio of short chains (DP 6–12) amylopectin (**Figure 5**). This is in good agreement with an increased proportion of intermediate chains observed in either *sbeIIb* mutant generated by chemical or physical mutagenesis or *sbeIIb* RNAi lines (Mizuno et al., 1993; Nishi et al., 2001; Satoh et al., 2003; Butardo et al., 2011; Zhu et al., 2012). As for *sbeI* mutant lines, a slight increase in the ratio of amylopectin short chains (DP 6–12) accompanied by a decrease in the long chains (DP > 14) was observed (**Figure 5**), consistent with findings from the previous *sbe1* rice mutant, which was generated through chemical mutagen, and had an altered fine structure of amylopectin caused by a significant decrease in chains of DP 12 to 21, and an increase in DP < 12 (Satoh et al., 2003). However, our results further indicated that rice *SBEI* plays a different role compared to *SBEI* genes in potato, maize, and wheat, in which the inactivation of *SBEI* resulted in no major impact on amylopectin structure and starch properties (Safford et al., 1998; Blauth et al., 2002; Regina et al., 2004).

It has been demonstrated that the higher proportion of long chains of amylopectin in starch granules led to resistance to gelatinization (Bhattacharyya et al., 1990; Mizuno et al., 1993; Stinard et al., 1993; Safford et al., 1998; Wang et al., 1998; Jane et al., 1999; Nishi et al., 2001). Our results showed that the onset, peak, and end temperature of thermo-gelatinization of the endosperm starches of *sbeIIb* mutants were significantly increased compared to those of wild type (**Table 3**). In contrast, the endosperm starches from the *sbeI* mutants had similar onset, peak, and end temperatures with those of wild type (**Table 3**), indicating that not only the increased of AC led to the suppression of hydration and swelling of starch, but also the modification of amylopectin fine structure affects physicochemical properties of starch. Furthermore, compared with wild type, an apparent increased RS was observed in the *sbeIIb* mutant lines (**Figure 7**). Moreover, the *sbeIIb* mutant lines also had an increased content of pentosan and decreased level of reducing sugar in comparison to wild type (**Table 3** and **Figure 7**), as observed in *SBEIIb* RNAi lines in rice (Butardo et al., 2011). Interestingly, slight differences existed in the RVA profiles between *sbeIIb-c1* and those of the rest *sbeIIb* lines (**Figure 6**), and RS contents between *sbeIIb-c3* and the rest of *sbeIIb* mutant lines (**Figure 7**), as observed in the fragrant rice generated by TALEN technology (Shan et al., 2015). The reason for this remains unclear; however, it implies that it is important to screen the mutant lines with the highest RS content and lowest reducing sugar content in a breeding program. Finally, it is worth to note that although the altered the fine structure of amylopectin and slight decreased resistance to thermo-gelatinization were observed in our *sbeI* mutant lines and in a previous *sbeI* mutant (Satoh et al., 2003), the proportion of RS and level of reducing sugar remained at levels similar to those of wild type (**Table 3** and **Figure 7**). These results suggest that except for amylose, longer and intermediate chain amylopectin molecules may also have an effect on property of the starch molecules, especially the elevations in the ratio of intermediate chains (DP > 14) are associated with increased level of RS in rice (Butardo et al., 2011).

Taken together, we here not only demonstrated the feasibility to create transgene-free high amylose rice plants through CRISPR/Cas9-mediated editing of *SBEIIb*, but also further defined that *SBEIIb* plays a more important role than *SBEI* in determining the fine structure of amylopectin and nutritional properties of starch in rice grain, and thus could be employed as a primary target gene for generating high-amylose rice to meet the increasing demand from people affected by diet-related non-infectious chronic diseases.

## Author Contributions

LX and YZ conceived and designed the experiments; YS, GJ, ZL, XZ, JL, XG, WD, and JD performed the experiments; YS and GJ analyzed the data; LX and YS wrote the manuscript; YZ and FF revised the manuscript. All authors read and approved the final manuscript.

## Conflict of Interest Statement

The authors declare that the research was conducted in the absence of any commercial or financial relationships that could be construed as a potential conflict of interest.

## Footnotes

1 http://dsdecode.scgene.com/

2 http://www.megazyme.com

3 http://www.beckmancoulter.com/

4 http://crispr.dbcls.jp/
