# Supplementary material for: Generation of High-Amylose Rice through CRISPR/Cas9-Mediated Targeted Mutagenesis of Starch Branching Enzymes
Source: Front Plant Sci. 2017 Mar 7;8:298. doi: 10.3389/fpls.2017.00298 (PMC5339335; doi:10.3389/fpls.2017.00298)
Supplement: Supplementary file 1 [file Table_1.DOCX]

**Generation of high-amylose rice through CRISPR/Cas9-mediated targeted mutagenesis of starch branching enzymes**

Yongwei Sun, Guiai Jiao, Zupei Liu, Xin Zhang, Jingying Li, Xiuping Guo, Wenming Du, Jinlu Du, Frédéric Francis, Yunde Zhao^*^ and Lanqin Xia ^*^

^*^Corresponding authors: Lanqin Xia, E-mail: [xialanqin@caas.cn](mailto:xialanqin@caas.cn) or Yunde Zhao, E-mail: yundezhao@ucsd.edu

**Supplementary Data**

**Table of contents**

1. **Supplementary Table 1. Analysis of potential off-target effects**
2. **Supplementary Table 2. Primers used in this study**

**Supplementary Table 1. Analysis of potential off-target effects**

| Target | Name of putative off-target site | Putative off-target locus | Sequence of the putative off-target site | No. of  mismatching bases | No. of plants sequenced | No. of plants with mutations |
| --- | --- | --- | --- | --- | --- | --- |
| *SBEI* | OFF1 | chr01:85288-85310 | CCCCGCCCGCGCCGCTTCTTCTC | 3 | 40 | 0 |
|  | OFF2 | chr01:1203350-1203372 | CCGCGCCCGCGCCCCTTCTTCCC | 3 | 40 | 0 |
|  | OFF3 | chr03:20750606-20750628 | CCTCCCCCTCTCCGCCCCTTCCC | 3 | 40 | 0 |
| *SBEIIb* | OFF4 | chr09:16385288-16385309 | CCTGCCTTAGATTATGAATTTAT | 3 | 31 | 0 |
|  | OFF5 | chr03:13608316-13608337 | CCAGCTTTAGATGTT_AATTAAG | 3 | 31 | 0 |

The PAM motif (NGG) is underlined; mismatching bases are shown in red.

**Supplementary Table 2. Primer sets used in this study**

| **Primer sets** | **Sequence (5′ To 3′)** | **Use of PCR products** | **Annealing (℃)** | **Fragment size (bp)** |
| --- | --- | --- | --- | --- |
| SBEI-F1 | AGGGAAGGAGCGGAGCGGGCGTTTTAGAGCTAGAAATAGCAAGTTA | Construct vector for gRNA1 | 60 | 146 |
| hrpme-u3R | CTGTCAAACACTGATAGTTTGGATCCTCTAGAGATTATG |  |  |  |
| hrpme-u3F | GTCGTTTCCCGCCTTCAGTTTTGCATGCCTGCAGGTCGACG | Construct vector for gRNA1 | 60 | 445 |
| SBEI-R1 | CGCCCGCTCCGCTCCTTCCCTGCCACGGATCATCTGCACAACTC |  |  |  |
| SBEIIb-F1 | ACTTAATTCATCATCTAAGGCTTTTAGAGCTAGAAATAGCAAGTTA | Construct vector for gRNA2 | 60 | 146 |
| hrpme-u3R | CTGTCAAACACTGATAGTTTGGATCCTCTAGAGATTATG |  |  |  |
| hrpme-u3F | GTCGTTTCCCGCCTTCAGTTTTGCATGCCTGCAGGTCGACG | Construct vector for gRNA2 | 60 | 445 |
| SBEIIb-R1 | GCCTTAGATGATGAATTAAGTGCCACGGATCATCTGCACAACTC |  |  |  |
| hrpme-u3F | GTCGTTTCCCGCCTTCAGTTTTGCATGCCTGCAGGTCGACG | Construct vector for gRNA1and gRNA2 | 60 | 570 |
| hrpme-u3R | CTGTCAAACACTGATAGTTTGGATCCTCTAGAGATTATG |  |  |  |
| RC11-F | CGCTATAAATCGCCGCC | Detect mutations at *SBEI* target site | 57 | 301 |
| RC11-R | GCGGCGAAGAAACCACG |  |  |  |
| RC33-F | TTAGTCCATACTAGTTGTCTGCGTG | Detect mutations of *SBEIIb* target site | 56 | 303 |
| RC33-R | TCAGCAGCTAATTCTTCAACCACTC |  |  |  |
| Cas9-F | TCGACAAGAAGTACTCCATCGGC | Detect Cas9 transgene in plants | 58 | 738 |
| Cas9-R | CAAGAGAGAGGGCGATCAGGTTG |  |  |  |
| U3F | GTAATTCATCCAGGTCTCCAAG | Detect gRNAs sequence in plants | 58 | 461 |
| U3R | ACTTTTTCAAGTTGATAACGG |  |  |  |
| ActinF | TGCTGACAGGATGAGCAAGG | Detect *actin* gene in plants | 55 | 690 |
| ActinR | CCCAACCATGCAAAGCTCAC |  |  |  |
| hptF | GAGGGCGTGGATATGTCCTG | Detect *hptII* gene in plants | 55 | 306 |
| hptR | ATTGACCGATTCCTTGCGGT |  |  |  |
| OFRC11-1F | GCAAAAACGGAAAGCCAAATGT | Detect mutations on putative off-target site of *SBEI* gRNA target | 58 | 317 |
| OFRC11-1R | GCCGCCATCTAGGAGGAGAT |  |  |  |
| OFRC11-2F | GTCCAGTGGAAGGTGTTCGT | Detect mutations on putative off-target site of *SBEI* gRNA target | 57 | 340 |
| OFRC11-2R | AACCAGCGGATACTTTGCGA |  |  |  |
| OFRC11-3F | TATGCCCCCTCTGTCCAAAC | Detect mutations on putative off-target site of *SBEI* gRNA target | 58 | 415 |
| OFRC11-3R | GAGGGGCGGCTCTAGATTTG |  |  |  |
| OFRC33-1F | ACTGCTGGTCAGTCAACCAT | Detect mutations on putative off-target site of *SBEIIb* gRNA target | 56 | 538 |
| OFRC33-1R | CGCACTACAGAGCAACGAGA |  |  |  |
| OFRC33-2F | CTCCAATGTTCCAGCACCGA | Detect mutations on putative off-target site of *SBEIIb* gRNA target | 59 | 521 |
| OFRC33-2R | CCGGGAACACATCTGGGTAG |  |  |  |
